# Supplementary material for: Missense Variants of von Willebrand Factor in the Background of COVID-19 Associated Coagulopathy
Source: Genes (Basel). 2023 Feb 28;14(3):617. doi: 10.3390/genes14030617 (PMC10048626; doi:10.3390/genes14030617)
Supplement: Supplementary file 1 [file genes-14-00617-s001.zip › genes-2183443-supplementary/supptable s1.pdf]

Supplementary Table S1. Haplotype frequencies of the *ADAMTS13* rs34024143, rs28729234, rs2301612 and *vWF* rs1800383, rs216311, rs216321, rs1063856, rs1800378 SNPs defined and calculated by HaploView v4.2

	**all (n = 72)**		**4 (n = 10)**		**5 (n = 35)**		**6 or 7 (n = 10)**		**8 or 9 (n = 17)**	
	**halpotype**	**frequency**	**halpotype**	**frequency**	**halpotype**	**frequency**	**halpotype**	**frequency**	**halpotype**	**frequency**
**ADAMTS13**	CCC	0.553	CCC	0.625	CCC	0.515	CCC	0.525	CCC	0.532
	CCG	0.394	CCG	0.281	CCG	0.441	CCG	0.475	CCG	0.406
	TTG	0.026	TTC	0.094	GGG	0.044			TCG	0.031
	TTC	0.020							TTG	0.031
	TCG	0.007								
**von Willebrandt factor**	CCCTC	0.232	CCCTC	0.448	CTCTC	0.166	CCCTC	0.278	CCCTC	0.425
	CCCTT	0.136	CTCCT	0.125	CCCTC	0.163	CCTTT	0.167	CTTTT	0.150
	CTCTC	0.109	CCCTT	0.115	CTCCC	0.144	CTCCT	0.167	CTCCC	0.102
	CCCCC	0.085	CCCCC	0.084	CCCTT	0.134	CTCTC	0.111	CCCCC	0.068
	CTCCC	0.080	CTTTT	0.063	CCTTC	0.092	CTCCC	0.111	CCCCT	0.056
	CTCCT	0.069	GTCCC	0.031	CTTCT	0.057	GCCTC	0.056	CTCCT	0.055
	CTTTT	0.063	CCTCC	0.031	CTTTT	0.057	CTTTT	0.056	CCCTT	0.040
	CCTTC	0.060	CTTCC	0.031	CTTTC	0.048	GCCCT	0.054	CCTTT	0.037
	CTTCT	0.037	CCTTT	0.031	CCTCT	0.037			CTCTT	0.036
	CTTTC	0.033	GCCCT	0.031	CCCCC	0.034			GCTCT	0.031
	CCTTT	0.022	CCCCT	0.010	GCCCC	0.026				
	GCCCC	0.018			CCCCT	0.024				
	GCCCT	0.018			GCTTC	0.018				
	CCTCT	0.015								
	GCCTC	0.011								
	GCTTC	0.007								
	CTTCC	0.003								
	CCTCC	0.002								
