# Supplementary material for: Missense Variants of von Willebrand Factor in the Background of COVID-19 Associated Coagulopathy
Source: Genes (Basel). 2023 Feb 28;14(3):617. doi: 10.3390/genes14030617 (PMC10048626; doi:10.3390/genes14030617)
Supplement: Supplementary file 1 [file genes-14-00617-s001.zip › genes-2183443-supplementary/supptable s2.pdf]

Supplementary Table S2. Genetic association between laboratory parameters and the investigated SNPs. As in extension of Table 6 Panel A, ANOVA analyses with non-significant results are shown.

	ADAMTS13			vWF				
	**rs2301612**	**rs28729234**	**rs34024143**	**rs1800383**	**rs216311**	**rs216321**	**rs1063856**	**rs1800378**
treatment in hospital (days)	0.486	0.845	0.845	0.918	0.196	0.694	0.705	0.741
treatment in intensive care unit (days)	0.653	0.496	0.642	0.936	0.075	0.500	0.964	0.874
lobar involvement (No)	0.109	0.406	0.450	0.876	0.478	0.753	0.137	0.876
respiratory rate (1/min)	0.379	0.638	0.521	0.966	0.866	0.597	0.575	0.974
Horowitz coefficient (PaO_2/FiO_2 Hgmm)	0.470	0.981	0.981	0.723	0.633	0.961	0.529	0.734
interleukin-6 (IL-6) (pg/ml)	0.110	0.121	0.256	0.929	0.248	0.612	0.267	0.699
C reactive protein CRP (mg/l)	0.539	0.991	0.918	0.440	0.730	0.868	0.427	0.383
D-dimer (mg/l)	0.152	0.855	0.952	0.219	0.895	0.428	0.533	0.469
